# Short- versus long-course antibiotic therapy for sepsis: a post hoc analysis of the nationwide cohort study

**DOI:** 10.1186/s40560-022-00642-3

**Published:** 2022-10-29

**Authors:** Nozomi Takahashi, Taro Imaeda, Taka‑aki Nakada, Takehiko Oami, Toshikazu Abe, Yasuo Yamao, Satoshi Nakagawa, Hiroshi Ogura, Nobuaki Shime, Asako Matsushima, Kiyohide Fushimi

**Affiliations:** 1 Department of Emergency and Critical Care Medicine, Chiba University Graduate School of Medicine, 1-8-1 Inohana, Chuo, Chiba 260-8677 Japan; 2 Health Services Research and Development Center, University of Tsukuba, Tsukuba, Japan; 3 Department of Emergency and Critical Care Medicine, Tsukuba Memorial Hospital, Tsukuba, Japan; 4 Department of Critical Care Medicine, National Center for Child Health and Development, Tokyo, Japan; 5 Department of Traumatology and Acute Critical Medicine, Osaka University Graduate School of Medicine, Osaka, Japan; 6 Department of Emergency and Critical Care Medicine, Graduate School of Biomedical and Health Sciences, Hiroshima University, Hiroshima, Japan; 7 Department of Emergency and Critical Care, Nagoya City University Graduate School of Medical Sciences, Aichi, Japan; 8 Department of Health Policy and Informatics, Tokyo Medical and Dental University Graduate School of Medical and Dental Sciences, Tokyo, Japan

**Keywords:** Sepsis, Antibiotics, Duration of treatment, Short-course

## Abstract

**Background:**

The appropriate duration of antibiotic treatment in patients with bacterial sepsis remains unclear. The purpose of this study was to evaluate the association of a shorter course of antibiotics on 28-day mortality in comparison with a longer course using a national database in Japan.

**Methods:**

We conducted a post hoc analysis from the retrospective observational study of patients with sepsis using a Japanese claims database from 2010 to 2017. The patient dataset was divided into short-course (≤ 7 days) and long-course (≥ 8 days) groups according to the duration of initial antibiotic administration. Subsequently, propensity score matching was performed to adjust the baseline imbalance between the two groups. The primary outcome was 28-day mortality. The secondary outcomes were re-initiated antibiotics at 3 and 7 days, during hospitalization, administration period, antibiotic-free days, and medical cost.

**Results:**

After propensity score matching, 448,146 pairs were analyzed. The 28-day mortality was significantly lower in the short-course group (hazard ratio, 0.94; 95% CI, 0.92–0.95; *P* < 0.001), while the occurrence of re-initiated antibiotics at 3 and 7 days and during hospitalization were significantly higher in the short-course group (*P* < 0.001). Antibiotic-free days (median [IQR]) were significantly shorter in the long-course group (21 days [17 days, 23 days] vs. 17 days [14 days, 19 days], *P* < 0.001), and short-course administration contributed to a decrease in medical costs (coefficient $-212, 95% CI; − 223 to − 201, *P* < 0.001). Subgroup analyses showed a significant decrease in the 28-day mortality of the patients in the short-course group in patients of male sex (hazard ratio: 0.91, 95% CI; 0.89–0.93), community-onset sepsis (hazard ratio; 0.95, 95% CI; 0.93–0.98), abdominal infection (hazard ratio; 0.92, 95% CI; 0.88–0.97) and heart infection (hazard ratio; 0.74, 95% CI; 0.61–0.90), while a significant increase was observed in patients with non-community-onset sepsis (hazard ratio; 1.09, 95% CI; 1.06–1.12).

**Conclusions:**

The 28-day mortality was significantly lower in the short-course group, even though there was a higher rate of re-initiated antibiotics in the short course.

**Supplementary Information:**

The online version contains supplementary material available at 10.1186/s40560-022-00642-3.

## Background

Antibiotic therapy is a key component in bacterial sepsis treatment; therefore, the appropriate choice of antibiotic spectrum and duration of administration are associated with the prognosis of sepsis and septic shock [1, 2]. Recently, several studies revealed that longer administration of antibiotics for sepsis was associated with an increase in antibiotic-resistant bacteria, episodes of *Clostridioides difficile* infection, and colonization of fungi, which could elevate the risk for recurrent sepsis [3, 4]. Accordingly, the determination of the optimal duration of antibiotics is important for the suppression of antimicrobial resistance, leading to improved outcomes in patients with sepsis [5, 6].

Previous studies have reported that in comparison with a longer course, a shorter course of antibiotics showed no difference in terms of mortality and treatment failure in several infectious foci [6, 7]. However, these studies had limited clinical application because of the small sample size or specific infection focus. Furthermore, while previous studies have covered infection, there are no studies on sepsis. For these reasons, longer use of antibiotics is often observed despite the recommendation for a shorter course in the real world [8, 9]. Furthermore, the influence of shorter antibiotic use, including recurrent infection or medical costs, was not evaluated in these studies. Due to the lack of high-quality evidence on sepsis, the superiority of shorter course of antibiotics over a longer course remains controversial, which leads to a weak recommendation for shorter duration of antibiotics with “very low quality of evidence” in the latest sepsis guidelines [1].

The aim of this study was to verify the hypothesis that a shorter course of antibiotics for patients with bacterial sepsis does not significantly increase 28-day mortality in comparison with a longer course, using a national database in Japan. Analysis of each focus of infection was performed considering clinical application. We also evaluated the influence of antibiotic duration on re-initiated antibiotics and medical costs.

## Methods

### Study design and data source

We conducted a post hoc analysis from the retrospective observational study of sepsis database which was extracted from the Japanese Diagnosis Procedure Combination (DPC) system from 2010 to 2017 [10]. DPC is a large administrative claims dataset of the national reimbursement system adopted in 71.5% of the acute care hospitals and covers more than 90% of the tertiary care emergency hospitals in Japan [11, 12]. This database consists of the data of approximately 7 million inpatients per year, and registers patients’ information, including primary diagnosis and comorbidities at admission or post-admission using the International Classification of Diseases, 10th Revision (ICD-10) codes, and the procedure code. The procedure code, including codes for mechanical ventilation and renal replacement therapy, was originally defined in Japanese [13, 14]. Since all medicines prescribed to hospitalized patients are also registered on a daily basis, the total or consecutive use days could be extracted from the database. This study was approved by the Institutional Review Board of Chiba University Graduate School of Medicine and was performed in accordance with the committee’s guidelines (approval number, 3429). The requirement to obtain informed consent was waived.

### Definition and date collection

Patients with sepsis were identified using the definition satisfying the condition of having both presumed serious infections and concurrent acute organ dysfunction, which is adapted from the present sepsis criteria [15, 16]. Presumed serious infection was defined using blood culture test records and antibiotic administration. The database of patients with sepsis using this definition has been previously reported [10].

Patients aged < 18 years or with missing data were excluded. Patients who survived over 7 days after hospitalization were included in the analysis to avoid the inclusion of patients who died within 7 days into the short-course group. Patients who survived 3 days or more were included in this since patients who received antibiotics less than 3 days were already excluded in previous study. Patients who required antibiotic administration of more than 14 days were also excluded to negate the influence of such as a difficulty of focus control and evaluate the adequate days of antibiotic administration [17–19]. Further details have been provided in the subsequent sections.

Age, sex, years of hospitalization, comorbidities, focus of infection, organ dysfunction, community-onset sepsis, first hospitalization, and intensive care unit (ICU) stay were extracted as baseline characteristics. In accordance with a previous report, comorbidities included malignant tumors, hypertension, diabetes mellitus, heart failure, cerebrovascular disease, chronic respiratory disease, ischemic heart disease, and chronic renal failure. To analyze the severity, acute organ dysfunction was defined in the following manner in accordance with a previous study [20]: “respiration” required mechanical ventilator within 2 days of sepsis onset, “cardiovascular function” required vasopressor within 2 days of sepsis onset, “renal” was registered with ICD-10 code indicating renal dysfunction and required diuretics or renal replacement therapy, and “coagulation” and “liver” were registered with ICD-10 code indicating dysfunction. While the DPC database does not include clinical information for severity scores, a previous study based on the DPC system revealed that these methods using ICD-10 codes for organ dysfunction systems reflect severity and mortality [21]. Neurological dysfunction was not extracted for analysis due to the difficulty of the detection using ICD-10 codes. Data regarding the length of hospital and ICU stays and duration of antibiotic administration were also extracted.

The short-course group was defined as patients in whom consecutive infusion of initial antibiotics was administered for 7 days or less, and the next infusion was administered 24 h after the end of the previous administration. Patients who received antibiotics through oral administration were not included in the study. The long-course group included patients who received a continuous infusion of antibiotics for more than 7 days. This threshold was set according to previous studies, in which the definition of “long-course” tended to be over 8 days [22–25]. Further, the patients who had end-stage kidney disease and accordingly received different administration methods, such as alternate-day dosing were excluded from the study cohort in the initial report [10]. In both groups, the first antibiotic infusion course was used for the analysis. Re-initiation of antibiotics at day 3 and 7 and during hospitalization after discontinuation were defined using the reappearance of ICD-10 code for antibiotics with an interval of each period after the last administration of initial antibiotics. For instance, a patient whose antibiotics stopped at 6 days, but were restarted at 9 days, would be considered to re-initiated antibiotics at 3 days. Community-onset sepsis was defined as sepsis within 48 h of hospitalization and remaining cohort was defined as hospital sepsis. First hospitalization was extracted as a characteristic when patients were hospitalized multiple times [10]. Medical cost was calculated using the summary of medical fee, which included medical procedure, fee of drugs, and medical material cost. The cost in Japanese yen was converted into U.S. dollars in accordance with the latest exchange rate on Aug 16, 2022 (134 yen = $1 USD).

### Outcomes

The primary outcome was 28-day mortality. Secondary outcomes were re-initiation of antibiotics at day 3 and 7 and during hospitalization after discontinuation, characteristics of patients with re-initiated antibiotics in hospitalization, antibiotic-free days, hospital length of stay and medical cost. Antibiotic-free days were calculated by the difference between hospital length of stay and administration period of antibiotics. We performed subgroup analysis by age, sex, focus of infection, presence or absence of community-onset sepsis, first hospitalization, and ICU stay for 28-day mortality. This analysis was also performed in respiratory, abdominal and urogenital infection. Base data for each analysis were set at sepsis onset. The threshold value of age was calculated using ROC curve analysis for 28-day mortality, and 75 years of age was used to maximize the sum of sensitivity and specificity.

### Statistical analysis

Continuous variables are presented as medians with interquartile ranges. Categorical variables were presented as numbers and percentages. Propensity score analyses were conducted to adjust for differences in baseline characteristics between the short- and long-course groups. We estimated the propensity of each patient to receive short-course administration using a logistic regression model, including each factor of the baseline characteristics. One-to-one propensity score matching using the nearest neighbor was performed without replacement, and the caliper width was set at 20% of the standard deviation of the propensity scores. Differences between groups before and after propensity score matching were assessed using standardized differences. A standardized difference of less than 0.1 suggests adequate variable balance after propensity matching, and a two-sided *P* value < 0.01 was considered statistically significant. The Mann–Whitney *U* test was used for comparing continuous variables, and Fisher’s exact test or Chi-square test was used for comparing categorical variables before and after propensity score matching. Kaplan–Meier curves were constructed for the primary outcome and compared using the log-rank test. Cox regression analysis was used for the subgroup analysis of the propensity score-matched cohort. Multiple regression analysis was used for the medical cost analysis using previously reported variables [26, 27]. Evaluation of the adequate study period was performed using sensitivity analysis where a cohort that patients registered from 2012 to 2016 was considered. This negated the influence of the change in the sepsis guideline which recommended different strategy for duration of antibiotics. Analyses were performed using R version 4.1.2 (R Foundation for Statistical Computing, Vienna, Austria, https://www.r-project.org/) and PRISM version 8 (GraphPad Software, Inc., La Jolla, California, USA).

## Results

**Fig. 1:**
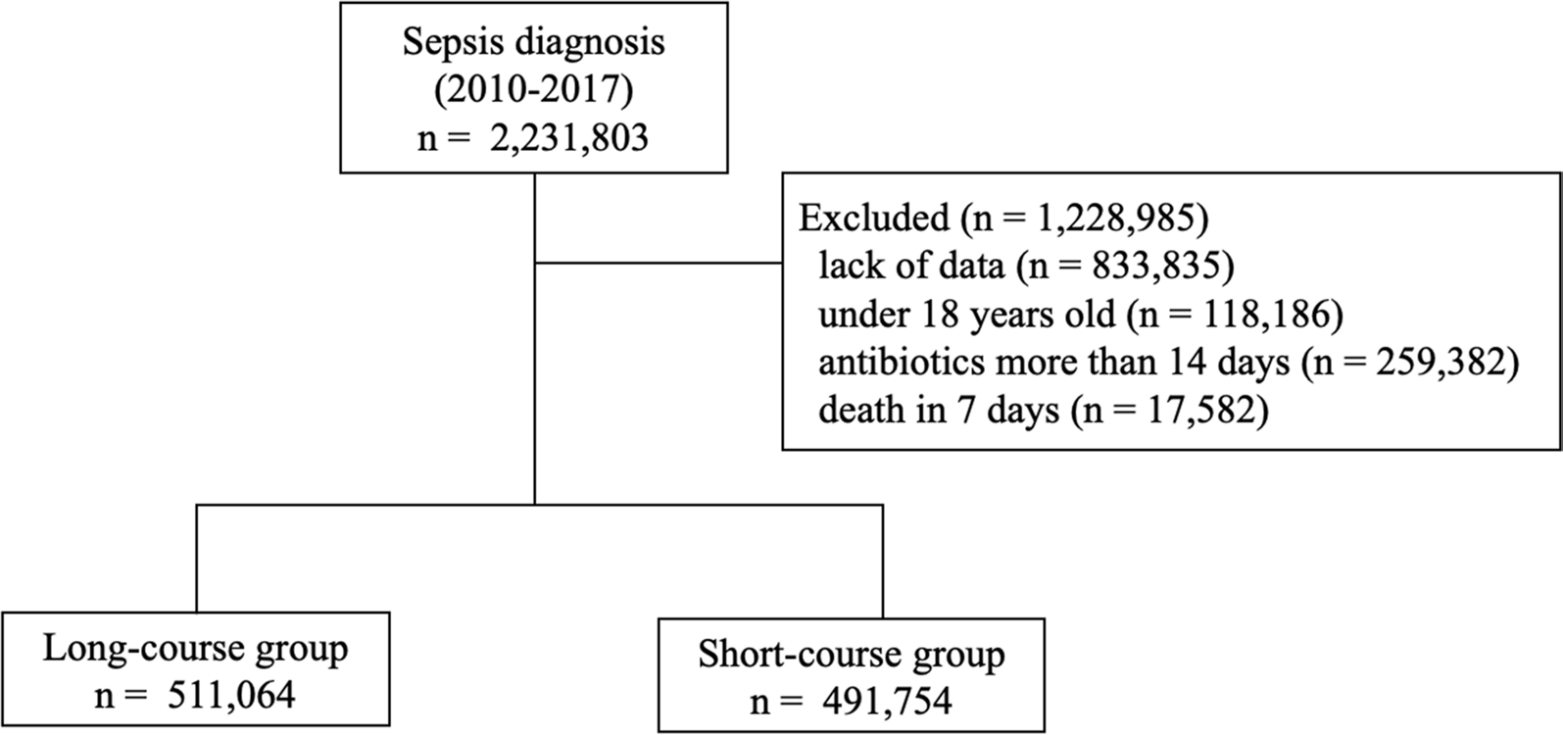
Flowchart of study population

Among 2,231,803 patients who met the sepsis criteria between 2010 and 2017, 1,228,985 were excluded due to lack of data, the age under 18 years, receiving antibiotics more than 14 days and death in 7 days, accordingly, 1,002,818 were included in the analysis (Fig. 1). In terms of baseline characteristics, the short-course group was more likely to be younger and female, have malignant tumors and ischemic heart disease, first hospitalization, and ICU stay (Additional file 1: Table S1). After propensity score matching, 448,146 pairs were generated, and standardized differences were < 0.1 for all variables of the matched cohort.

### Outcomes

**Fig. 2:**
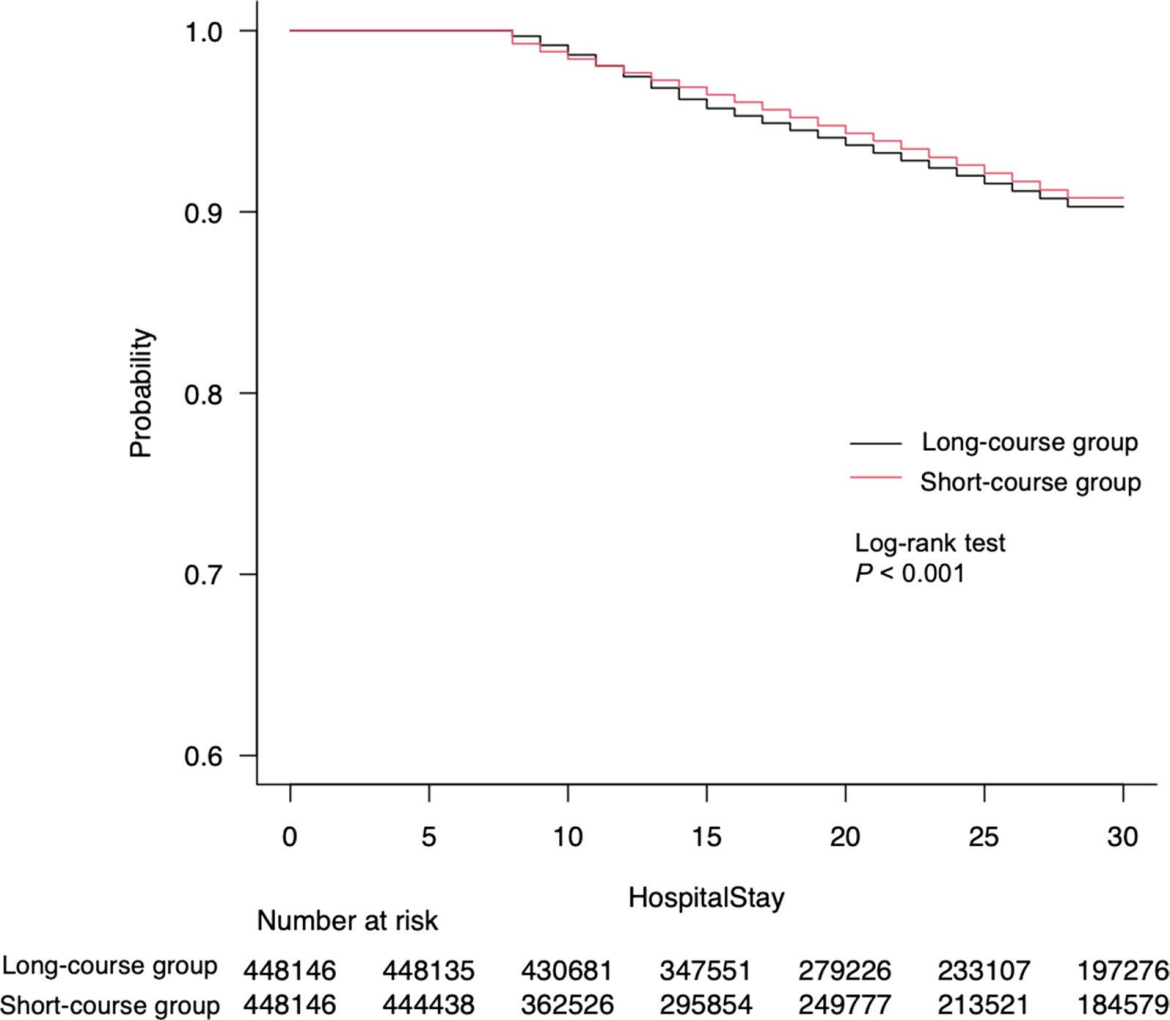
Kaplan–Meier curve for 28-day mortality between short and long-course group. There was a significant difference between the two groups (log-rank test, *P* < 0.001)

The primary endpoint of 28-day mortality occurred in 26,804 of 448,146 patients in the short-course group (6.0%), as compared with 32,753 of 448,146 patients in the long-course group (7.3%) (hazard ratio [HR], 0.94; 95% confidence interval [CI]; 0.92–0.95, *P* < 0.001). Kaplan–Meier analysis showed a significant difference between the two groups in the time to the primary outcome (log-rank test, *P* < 0.001) (Fig. 2).

In the secondary outcomes, there were significant differences between the two groups in re-initiated antibiotics within 3 days (short-course group: 19,453 patients [4.3%] and long-course group: 11,299 patients [2.5%], *P* < 0.001), re-initiated antibiotics within 7 days (short-course group: 67,284 patients [15.0%] and long-course group: 43,978 patients [9.8%], *P* < 0.001), and re-initiated antibiotics in hospitalization (short-course group: 148,412 patients [33.1%] and long-course group: 115,138 patients [25.7%]; *P* < 0.001) (Table 1). In the analysis of patients’ characteristics with re-initiated antibiotics in hospitalization, some factors including comorbidities of malignant tumor (non-reinitiated antibiotics group: 175,569 patients [27.7%] and re-initiated antibiotics group: 108,257 patients [41.1%], *P* < 0.001), cerebrovascular disease (non-reinitiated antibiotics group: 78,399 patients [12.4%] and re-initiated antibiotics group: 45,007 patients [17.1%], *P* < 0.001) and chronic renal failure (non-reinitiated antibiotics group: 76,772 patients [12.1%] and re-initiated antibiotics group: 21,249 patients [8.1%], *P* < 0.001), multifocal (non-reinitiated antibiotics group: 156,812 patients [24.8%] and re-initiated antibiotics group: 86,039 patients [32.6%], *P* < 0.001) and ICU admission (non-reinitiated antibiotics group: 30,838 patients [4.9%] and re-initiated antibiotics group: 23,033 patients [8.7%], *P* < 0.001) were associated with re-initiated antibiotics administration, whereas the comorbidity of chronic respiratory disease (non-reinitiated antibiotics group: 76,772 patients [12.1%] and re-initiated antibiotics group: 21,249 patients [8.1%], *P* < 0.001), respiratory sepsis (non-reinitiated antibiotics group: 244,676 patients [38.7%] and re-initiated antibiotics group: 69,428 patients [26.3%], *P* < 0.001) and community-onset sepsis (non-reinitiated antibiotics group: 446,450 patients [70.6%] and re-initiated antibiotics group: 97,368 patients [36.9%], *P* < 0.001) were associated with non-reinitiated antibiotics administration (Additional file 1: Table S2). The administration period of initial antibiotics was significantly shorter in the short-course group (short-course group: 5 days [4 days, 7 days] and short-course group: 10 days [9 days, 12 days], *P* < 0.001), and antibiotic-free days were significantly shorter in the long-course group (short-course group: 21 days [17 days, 23 days] and long-course group: 17 days [14 days, 19 days], *P* < 0.001). Hospital length of stay was significantly shorter in the short-course group (short-course group: 24 days [12 days, 50 days] and long-course group: 31 days [18 days, 55 days], P < 0.001). Medical costs were significantly higher in the long-course group than in the short-course group (short-course group: $8970 [$4412, $19,176] and long-course group: $9,766 [$5630, $18,261], *P* < 0.001). Short-course administration contributed to a decrease after adjusting for multiple variables (short-course group, coefficient $-212, 95% CI: − 223 to − 201, *P* < 0.001, adjusted R^2^ = 0.35) (Additional file 1: Table S3).

**Table 1:** Secondary outcomes after propensity score matching

Outcomes	Propensity score-matched cohort		*P* value
	Short-course group (*n* = 448,146)	Long-course group (*n* = 448,146)	
Re-initiated antibiotics in 3 days, *n* (%)	19,453 (4.3)	11,299 (2.5)	< 0.001
Re-initiated antibiotics in 7 days, *n* (%)	67,284 (15.0)	43,978 (9.8)	< 0.001
Re-initiated antibiotics in hospitalization, *n* (%)	148,412 (33.1)	115,138 (25.7)	< 0.001
Antibiotic-free days	21 (17, 23)	17 (14, 19)	< 0.001
Hospital length of stay, days	24 (12, 50)	31 (18, 55)	< 0.001
Medical cost, dollars	8,970 [4412, 19,176]	9,766 [5630, 18,261]	< 0.001

**Fig. 3:**
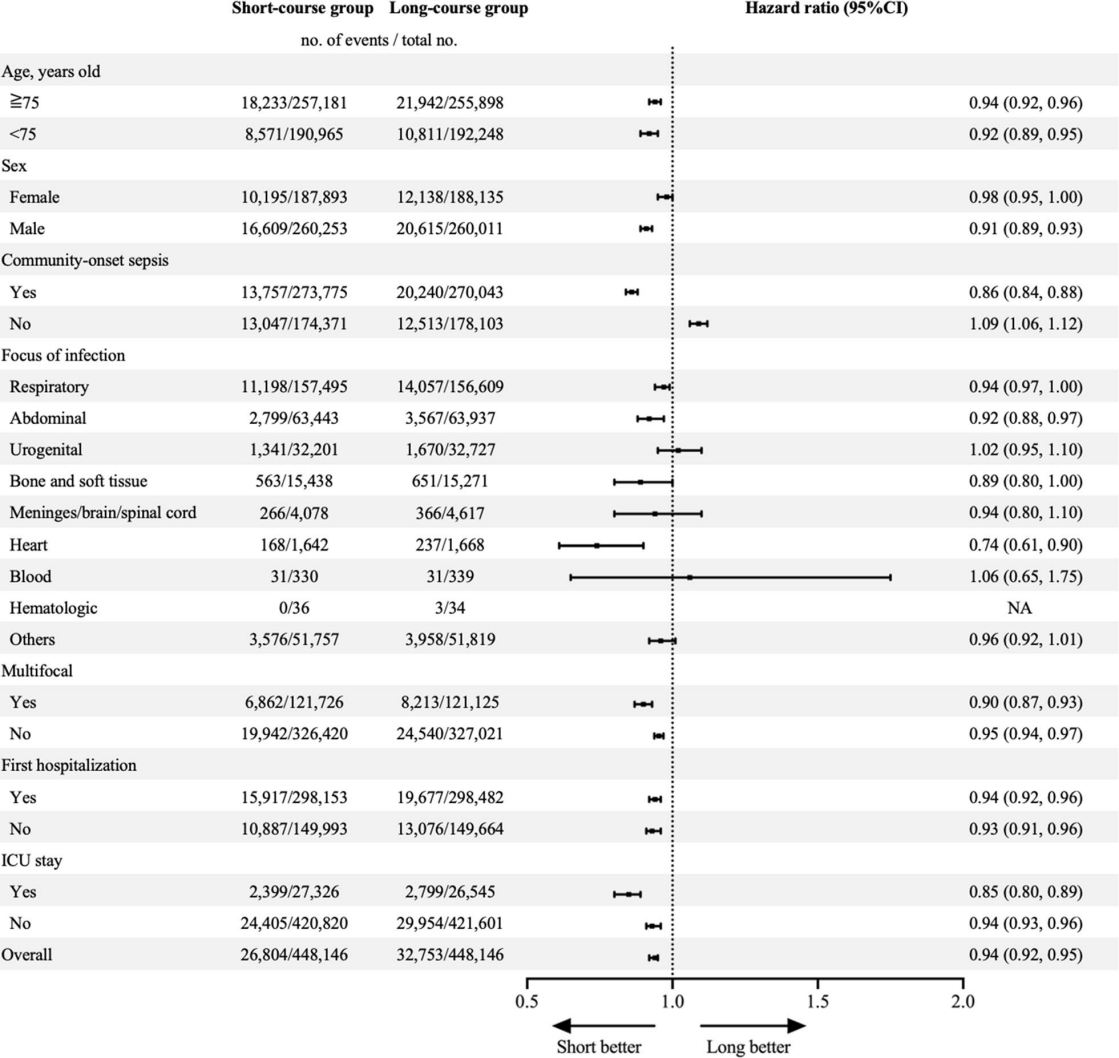
Subgroup analysis for 28-day mortality by Cox regression analysis. The hazard ratio is depicted as a circle with a 95% confidence interval in the table

Subgroup analyses for 28-day mortality showed significant differences between the two groups in male patients (short-course group: 16,609 of 260,253 patients and long-course group: 20,615 of 260,011 patients, HR; 0.91, 95% CI; 0.89–0.93), patients with community-onset sepsis (short-course group: 13,757 of 273,775 patients and long-course group: 20,240 of 270,043 patients, HR; 0.86, 95%CI; 0.84–0.88), patients with non-community-onset sepsis (short-course group: 13,047 of 174,371 patients and long-course group: 12,513 of 178,103 patients, HR; 1.09, 95% CI; 1.06–1.12), patients with abdominal infection (short-course group: 2799 of 63,443 patients and long-course group: 3567 of 63,937 patients, HR; 0.92, 95% CI; 0.88–0.97) and patients with heart infection (short-course group: 168 of 1,642 patients and long-course group: 237 of 1668 patients, HR; 0.74, 95% CI; 0.61–0.90) (Fig. 3).

As to each focus, short-course administration was associated with decreased 28-day mortality in patients with male (HR; 0.85, 95% CI; 0.79–0.92) and community-onset sepsis (HR; 0.85, 95% CI; 0.78–0.91) in abdominal infection, while these were not observed in respiratory and urogenital infection (Additional file 1: Table S5).

### Sensitivity analysis

In the cohort of the study period from 2012 to 2016 after propensity score matching, the 28-day mortality did not show significant deference between the short-course group and long-course group (long-course group: 33,450 of 331,485 [10.1%] and short-course group: 32,433 [9.8%], HR: 1.02, 95% CI; 0.99–1.04, *P* = 0.065) (Additional file 1: Table S3, Figure S1).

## Discussion

The observational nationwide study of 896,292 patients with sepsis demonstrated a significance for lower 28-day mortality in patients who received initial antibiotics for a duration of 7 days or less, while re-initiated antibiotics occurred marginally more significantly. The subgroup analysis indicated a significant association between the 28-day mortality and specific factors.

Randomized control trials (RCTs) investigating the efficacy of short-course antibiotic therapy have been performed with limited focus on infection [28–33]. Furthermore, few systematic reviews and meta-analyses have compared shorter and longer courses of antibiotics for infection, and no previous study has focused solely on sepsis other than critically ill patients [6, 34]. To the best of our knowledge, our study is the first to examine the associated difference in 28-day mortality between short- and long-course antibiotics duration in sepsis among different types of infection.

In this study, shorter antibiotic duration demonstrated a significance for lower 28-day mortality, although there was an increased rate of re-initiated antibiotics in all groups during the study period, which is similar to the result of a previous study on infection [6]. Furthermore, there were significant differences between the short- and long-course groups in subgroup analyses. The focus of infection is an important factor when considering the antibiotic duration, and several studies have revealed different results for mortality according to each focus [22–24, 35–38]. It is notable that the outcome was different between each type of infection in the subgroup analysis. A previous RCT comparing 3 days versus 8 days of antibiotic treatment for patients with community-acquired pneumonia revealed immutable clinical success rates on day 10 in the 3-day group [29]. Similarly, two RCTs for intra-abdominal infection among source-controlled patients showed non-inferiority of short-course antibiotic administration [32, 33]. Similar relationships were also found in our study focusing on sepsis, which may indicate the importance of source control, since drainage procedures tend to be more difficult for respiratory infections than for intra-abdominal infections. Furthermore, in the analysis for each focus, some factors indicated the significant difference for the mortality of long-course administration in abdominal infection. Accordingly, physicians should recognize the heterogeneity of infections by considering the duration of administration.

Our study also identified the importance of the classification of community-onset sepsis using subgroup analysis. No previous study has focused on community-onset sepsis or investigated the inferiority of short-course administration of antibiotics. The results of the present study may indicate that infectious organisms that cause community-onset sepsis do not tend to have antimicrobial resistance and require shorter antibiotic administration since non-resistant organisms might be easily driven out [39]. In contrast, patients with non-community-onset sepsis in the short-course group showed significantly higher mortality, indicating in-hospital sepsis. Previous RCTs on ventilator-associated pneumonia showed no significant difference in short-course mortality between the two groups, whereas our results of non-community-onset sepsis showed an inferior short-course duration [28, 30]. This may depend on organ dysfunction concerning the duration of antibiotics in non-community-onset sepsis, and further studies focusing on this are needed.

The mortality rate of female patients was not significantly different between two groups. A previous study focusing on patients with sepsis revealed that the female sex was significantly associated with hospital mortality after adjustment, although the number of dysfunctional organs did not differ by sex [40]. Another study reported that estrogens have physiological actions that could be detrimental to sepsis [41]. Sex differences in infection sites were also previously indicated [42, 43]; however, the effect on mortality by site was inconsistent. Further studies focusing on the association between sex, infection site, and disease severity are required since the personalized strategy should be important for clinical application.

Medical costs were significantly lower in the short-course group even after adjusting for baseline imbalances. Few studies have reported that a shorter course saved the cost of antibiotics in patients with spontaneous bacterial peritonitis [44, 45]; however, no investigation has examined the difference in medical costs associated with sepsis. As sepsis is an important contributor to the global disease burden, this result supports the shorter use of antibiotics in sepsis [46].

The sensitivity analysis which separated the study period between 2012 and 2016 showed no significant difference in terms of the mortality. In the primary analysis, the proportion of cases in 2010 and 2011 was lower than that of other periods. It may be explained by the change of the clinical guideline for sepsis management revised in 2012, which recommended to obtain blood culture and recognize the sepsis. Propensity score matching could not adjust this influence and the change of decision-making of the physician. Furthermore, cases increased to 35.8% in the long-course group, while these increased to 30.9% in the short-course group, although there has been a tendency to shorten the duration of antibiotic use in recent years. The period which was used for sensitivity analysis might reduce the effect of these problems; however, prospective study in which the management of sepsis is unified should be needed.

The present study had some limitations. First, this study is a retrospective study; therefore, unmeasured confounders may remain. For instance, the database does not include source control of infection. As stated above, source control is the key strategy for sepsis, and it affects the decision-making of antibiotic strategies for physicians. Although we could extract the surgical codes of the ICD-10, we did not use them for analysis because it was difficult to identify whether the procedure was performed for the source control of infection. This database also has the possibility of extracting non-septic patients due to the methodology which indirectly collected using ICD-10 codes corresponding the organ dysfunction, resulting in the low rate of mortality in the study. Second, the summary of Sequential Organ Failure Assessment (SOFA) score was not included to adjust the baseline imbalance in propensity score matching. However, the organ dysfunction of respiration and cardiovascular dysfunction we determined to extract severity in this study was set at 2 or 3 points in SOFA score and this method reflected severity and mortality in previous study [21]. Furthermore, short-course patients who were discharged before 7 days died due to disease severity or had viral infection, or longer course patients who required antibiotic administration of more than 14 days which was related to survival bias, were excluded from the study. The low rate of 28-day mortality in this study might be also related to the study cohort excluding the patients who dead in 7 days. Low fraction of ICU patients and lower mortality compared to the previous study might reflect these biases and it indicated the clinical application for severe case of sepsis. Third, patients with community-acquired pneumonia but not necessarily with associated sepsis may have been included as sepsis cases when they only had respiratory dysfunction since oxygen therapy alone counted as respiratory dysfunction. The high proportion of respiratory dysfunction in the study may reflect this possibility, while oxygen requirements generally increase in sepsis patients. Fourth, the onset day of the organ dysfunction used for the definition of sepsis in the study cohort did not necessarily equal as that of sepsis, except for the respiratory and cardiovascular dysfunction which was registered on a daily basis. It also means that other factors affected the propensity score matching, which may cause the study bias. Fifth, this study did not have the information of antibiotics. The difference of antibiotics type might affect the outcome. Finally, the antimicrobial susceptibility of the bacteria was not taken into consideration because the DPC system does not contain information on pathogenic bacteria. Further studies that include microorganism data and analyses of the association with the antimicrobial susceptibility are needed.

## Conclusions

The 28-day mortality was significantly lower in the short course, even though there was a higher rate of re-initiated antibiotics in the short course.

## Supplementary Information

**Additional file 1: Table S1.** Baseline characteristics. **Table S2.** Baseline characteristics for sensitivity analysis (2012 to 2016). **Table S3.** Patient characteristics between non-reinitiated or reinitiated antibiotics group. **Table S4.** Multiple regression analysis for medical cost. **Table S5.** Analysis for 28-day mortality in each focus by Cox regression analysis. **Figure S1.** Kaplan–Meier curve for 28-day mortality between short and long-course group in the cohort between 2012 and 2016

## Data Availability

The datasets analyzed during the current study are available with the corresponding author on reasonable request.

## Abbreviations

ICU: Intensive care unit
DPC: Diagnostic procedure combination
ICD-10: International Statistical Classification of Diseases and Related Health Problems 10th Revision
CI: Confidence interval
SOFA: Sequential Organ Failure Assessment
