# Supplementary material for: Short- versus long-course antibiotic therapy for sepsis: a post hoc analysis of the nationwide cohort study
Source: J Intensive Care. 2022 Oct 29;10:49. doi: 10.1186/s40560-022-00642-3 (PMC9617305; doi:10.1186/s40560-022-00642-3)

*Supplemental Digital Content*

**Short versus long course antibiotic therapy for sepsis: A nationwide cohort study**

**Authors:** Nozomi Takahashi, M.D., Ph.D.[1], Taro Imaeda, M.D., Ph.D.[1], Taka-aki Nakada, M.D., Ph.D.[1], Takehiko Oami, M.D., Ph.D.[1], Toshikazu Abe, M.D., Ph.D.[2,3], Yasuo Yamao[1], Satoshi Nakagawa, M.D., Ph.D.[4], Hiroshi Ogura, M.D., Ph.D.[5], Nobuaki Shime, M.D., Ph.D.[6], Asako Matsushima, M.D., Ph.D.[7] and Kiyohide Fushimi, M.D., Ph.D.[8]

**Table of Contents.**

**Supplementary Table 1. Baseline characteristics**
**Supplementary Table 2. Baseline characteristics for sensitivity analysis (2012 to 2016)**
**Supplementary Table 3. Patient characteristics between non-re-initiated or re-initiated antibiotics group**
**Supplementary Table 4. Multiple regression analysis for medical cost**
**Supplementary Table 5. Analysis for 28-day mortality in each focus by cox regression analysis**
**Supplementary Figure 1. Kaplan-Meier curve for 28-day mortality between short and long-course group in the cohort between 2012 and 2016**

**Supplementary Table 1. Baseline characteristics**

| characteristics | Full cohort | | | | Propensity score-matched cohort | | | |
|---|---|---|---|---|---|---|---|---|
| | Short-course group (n = 491,574) | Long-course group (n = 511,064) | *P* value | SMD | Short-course group (n = 448,146) | Long-course group (n = 448,146) | *P* value | SMD |
| Age, years | 76 (66, 84) | 77 (68, 85) | <0.001 | 0.12 | 77 (67, 84) | 76 (66, 84) | 0.069 | 0.005 |
| Male (%) | 284,490 (57.9) | 297,669 (58.2) | <0.001 | 0.008 | 260,253 (58.1) | 260,011 (58.0) | 0.606 | 0.001 |
| Year, n (%) | | | <0.001 | 0.050 | | | <0.001 | 0.115 |
| 2010 | 27,772 (5.6) | 26,563 (5.2) | | | 27,753 (6.2) | 24,156 (5.4) | | |
| 2011 | 41,055 (8.3) | 39,496 (7.7) | | | 41,024 (9.2) | 35,603 (7.9) | | |
| 2012 | 50,741 (10.3) | 49,411 (9.7) | | | 50,356 (11.2) | 44,627 (10.0) | | |
| 2013 | 55,643 (11.3) | 55,816 (10.9) | | | 55,336 (12.3) | 49,521 (11.1) | | |
| 2014 | 70,108 (14.3) | 72,498 (14.2) | | | 65,238 (14.6) | 64,020 (14.3) | | |
| 2015 | 74,299 (15.1) | 80,128 (15.7) | | | 70,043 (15.6) | 69,804 (15.6) | | |
| 2016 | 85,474 (17.4) | 92,413 (18.1) | | | 67,490 (15.1) | 79,876 (17.8) | | |
| 2017 | 86,662 (17.6) | 94,739 (18.5) | | | 70,906 (15.8) | 80,539 (18.0) | | |
| Comorbidity, n (%) | | | | | | | | |
| Malignant tumor | 161,184 (32.8) | 157,121 (30.7) | <0.001 | 0.044 | 141,680 (31.6) | 142,146 (31.7) | 0.291 | 0.002 |
| Hypertension | 129,030 (26.2) | 133,435 (26.1) | 0.14 | 0.003 | 116,803 (26.1) | 117,865 (26.3) | 0.011 | 0.005 |
| Diabetes mellitus | 103,808 (21.1) | 111,818 (21.9) | <0.001 | 0.019 | 96,184 (21.5) | 96,418 (21.5) | 0.549 | 0.001 |

| | | | | | | | | |
|---|---|---|---|---|---|---|---|---|
| Heart failure | 84,261 (17.1) | 99,864 (19.5) | <0.001 | 0.062 | 80,317 (17.9) | 81,021 (18.1) | 0.053 | 0.004 |
| Cerebrovascular disease | 67,711 (13.8) | 70,871 (13.9) | 0.16 | 0.003 | 61,433 (13.7) | 61,973 (13.8) | 0.098 | 0.003 |
| Chronic respiratory disease | 51,018 (10.4) | 57,610 (11.3) | <0.001 | 0.029 | 49,220 (11.0) | 48,801 (10.9) | 0.157 | 0.003 |
| Ischemic heart disease | 47,851 (9.7) | 48,545 (9.5) | <0.001 | 0.008 | 42,544 (9.5) | 43,124 (9.6) | 0.038 | 0.004 |
| Chronic renal failure | 17,458 (3.6) | 19,303 (3.8) | <0.001 | 0.012 | 16,425 (3.7) | 16,381 (3.7) | 0.809 | 0.001 |
| Focus of infection, n (%) | | | | 0.138 | | | | 0.014 |
| Respiratory | 161,871 (32.9) | 187,128 (36.6) | | | 157,495 (35.1) | 156,609 (34.9) | | |
| Abdominal | 80,346 (16.3) | 65,681 (12.9) | | | 63,443 (14.2) | 63,937 (14.3) | | |
| Urogenital | 59,306 (12.1) | 55,920 (10.9) | | | 32,201 (7.2) | 32,727 (7.3) | | |
| Bone and soft tissue | 16,187 (3.3) | 18,061 (3.5) | | | 15,438 (3.4) | 15,271 (3.4) | | |
| Meninges/brain/spinal cord, | 6,513 (1.3) | 4,658 (0.9) | | | 4,078 (0.9) | 4,617 (1.0) | | |
| Heart | 2,129 (0.4) | 1,715 (0.3) | | | 1,642 (0.4) | 1,668 (0.4) | | |
| Blood | 332 (0.1) | 478 (0.1) | | | 330 (0.1) | 339 (0.1) | | |
| Hematologic | 38 (0.0) | 36 (0.0) | | | 36 (0.0) | 34 (0.0) | | |
| Others | 59,306 (12.1) | 55,920 (10.9) | | | 51,757 (11.5) | 51,819 (11.6) | | |
| Multifocal | 126,746 (25.8) | 143,142 (28.0) | | | 121,726 (27.2) | 121,125 (27.0) | | |

| | | | | | | | | |
|---|---|---|---|---|---|---|---|---|
| Organ dysfunction, n (%) | | | | | | | | |
| Respiration | 345,690 (70.3) | 383,402 (75.0) | <0.001 | 0.106 | 319,737 (71.3) | 326,704 (72.9) | <0.001 | 0.035 |
| Coagulation | 47,432 (9.6) | 60,618 (11.9) | <0.001 | 0.072 | 46,701 (10.4) | 46,708 (10.4) | 0.983 | <0.001 |
| Cardiovascular function | 38,960 (7.9) | 35,470 (6.9) | <0.001 | 0.037 | 28,910 (6.5) | 32,918 (7.3) | <0.001 | 0.035 |
| Liver | 21,068 (4.3) | 19,216 (3.8) | <0.001 | 0.027 | 17,969 (4.0) | 17,791 (4.0) | 0.339 | 0.002 |
| Renal | 189,037 (38.4) | 203,321 (39.8) | <0.001 | 0.028 | 176,134 (39.3) | 174,329 (38.9) | <0.001 | 0.008 |
| Community-onset sepsis, n (%) | 282,658 (57.5) | 323,554 (63.3) | <0.001 | 0.119 | 273,775 (61.1) | 270,043 (60.3) | <0.001 | 0.017 |
| First hospitalization, n (%) | 331,655 (67.4) | 335,179 (65.6) | <0.001 | 0.039 | 298,153 (66.5) | 298,482 (66.6) | 0.463 | 0.002 |
| ICU stay, n (%) | 32,383 (6.6) | 28,059 (5.5) | <0.001 | 0.046 | 27,326 (6.1) | 26,545 (5.9) | 0.001 | 0.007 |

Data are presented as median (interquartile range).

SMD, Standardized mean difference; ICU, intensive care unit

**Supplementary Table 2. Baseline characteristics for sensitivity analysis (2012 to 2016)**

| | Full cohort | | | | Propensity score-matched cohort | | | |
|---|---|---|---|---|---|---|---|---|
| characteristics | Short-course group (n = 336,265) | Long-course group (n = 529,308) | *P* value | SMD | Short-course group (n = 331,485) | Long-course group (n = 331,485) | *P* value | SMD |
| Age, years | 76 (66, 84) | 77 (67, 84) | <0.001 | 0.069 | 76 (66, 84) | 76 (66, 84) | 0.058 | <0.001 |
| Male (%) | 194,576 (57.9) | 315,428 (59.6) | <0.001 | 0.035 | 192.652 (58.1) | 192,017 (57.9) | 0.12 | 0.004 |
| Year, n (%) | | | | 0.041 | | | | 0.022 |
| 2012 | 50,741 (15.1) | 74,266 (14.0) | | | 50,733 (15.3) | 49,161 (14.8) | | |
| 2013 | 55,643 (16.5) | 84,450 (16.0) | | | 55,640 (16.8) | 54,289 (16.4) | | |
| 2014 | 70,108 (20.8) | 109,500 (20.7) | | | 68,818 (20.8) | 70,015 (21.1) | | |
| 2015 | 74,299 (22.1) | 120,921 (22.8) | | | 73,338 (22.1) | 75,441 (22.8) | | |
| 2016 | 85,474 (25.4) | 140,171 (26.5) | | | 82,956 (25.0) | 82,579 (24.9) | | |
| Comorbidity, n (%) | | | | | | | | |
| Malignant tumor | 110,371 (32.8) | 174,262 (32.9) | 0.34 | 0.044 | 108,611 (32.8) | 108,693 (32.8) | 0.83 | 0.001 |
| Hypertension | 87,970 (26.2) | 131,850 (24.9) | <0.001 | 0.029 | 86,451 (26.1) | 86,818 (26.2) | 0.31 | 0.003 |
| Diabetes mellitus | 70,755 (21.0) | 117,067 (22.1) | <0.001 | 0.026 | 70,219 (21.2) | 69,983 (21.1) | 0.48 | 0.002 |
| Heart failure | 57,022 (17.0) | 100,335 (19.0) | <0.001 | 0.052 | 56,790 (17.1) | 56,986 (17.2) | 0.53 | 0.002 |
| Cerebrovascular disease | 46,176 (13.7) | 73,086 (13.8) | 0.32 | 0.002 | 45,524 (13.7) | 46,029 (13.9) | 0.073 | 0.004 |

| | | | | | | | | |
|---|---|---|---|---|---|---|---|---|
| Chronic respiratory disease | 35,077 (10.4) | 53,958 (10.2) | <0.001 | 0.008 | 34,882 (10.5) | 34,812 (10.5) | 0.78 | 0.001 |
| Ischemic heart disease | 32,741 (9.7) | 48,922 (9.2) | <0.001 | 0.017 | 32,106 (9.7) | 32,306 (9.7) | 0.41 | 0.002 |
| Chronic renal failure | 12,025 (3.6) | 20,730 (3.9) | <0.001 | 0.018 | 11,965 (3.6) | 12,076 (3.6) | 0.47 | 0.002 |
| Focus of infection, n (%) | | | | 0.19 | | | | 0.013 |
| Respiratory | 108,033 (32.1) | 171,849 (32.5) | | | 107,949 (32.6) | 106,929 (32.3) | | |
| Abdominal | 56,367 (16.8) | 65,392 (12.4) | | | 53,491 (16.1) | 54,177 (16.3) | | |
| Urogenital | 27,378 (8.1) | 31,163 (5.9) | | | 25,941 (7.8) | 26,540 (8.0) | | |
| Bone and soft tissue | 11,329 (3.4) | 20,402 (3.9) | | | 11,328 (3.4) | 11,278 (3.4) | | |
| Meninges/brain/spinal cord, | 4,668 (1.4) | 5,779 (1.1) | | | 4,488 (1.4) | 4,543 (1.4) | | |
| Heart | 1,490 (0.4) | 4,395 (0.8) | | | 1,490 (0.4) | 1,629 (0.5) | | |
| Blood | 235 (0.1) | 666 (0.1) | | | 235 (0.1) | 273 (0.1) | | |
| Hematologic | 30 (0.0) | 32 (0.0) | | | 28 (0.0) | 31 (0.0) | | |
| Others | 41,398 (12.3) | 63,934 (12.1) | | | 41,199 (12.4) | 40,924 (12.3) | | |
| Multifocal | 85,337 (25.4) | 165,696 (31.3) | | | 85,336 (25.7) | 85,161 (25.7) | | |
| Organ dysfunction, n (%) | | | | | | | | |
| Respiration | 230,778 (68.6) | 389,330 (73.6) | <0.001 | 0.11 | 229,838 (69.3) | 229,335 (69.2) | 0.18 | 0.003 |
| Coagulation | 32,434 (9.6) | 76,904 (14.5) | <0.001 | 0.15 | 32,433 (9.8) | 33,450 (10.1) | <0.001 | 0.01 |

| | | | | | | | | |
|---|---|---|---|---|---|---|---|---|
| Cardiovascular function | 25,207 (7.5) | 41,379 (7.8) | <0.001 | 0.012 | 24,304 (7.3) | 24,773 (7.5) | 0.028 | 0.005 |
| Liver | 14,574 (4.3) | 19,773 (3.7) | <0.001 | 0.030 | 13,794 (4.2) | 14,111 (4.3) | 0.053 | 0.005 |
| Renal | 128,115 (38.1) | 226,953 (42.9) | <0.001 | 0.097 | 127,650 (38.5) | 127,837 (38.6) | 0.64 | 0.001 |
| Community-onset sepsis, n (%) | 194,716 (57.9) | 305,019 (57.6) | 0.01 | 0.006 | 192,755 (58.1) | 191,993 (57.9) | 0.058 | 0.005 |
| First hospitalization, n (%) | 233,136 (69.3) | 359,672 (68.0) | <0.001 | 0.030 | 229,037 (69.1) | 229,227 (69.2) | 0.62 | 0.001 |
| ICU stay, n (%) | 17,169 (5.1) | 26,923 (5.1) | 0.69 | 0.001 | 16,764 (5.1) | 16,873 (5.1) | 0.55 | 0.001 |

Data are presented as median (interquartile range).

SMD, Standardized mean difference; ICU, intensive care unit

**Supplementary Table 3. Patient characteristics between non-re-initiated or re-initiated antibiotics group**

| characteristics | Non-re-initiated antibiotics group (n = 632,742) | Re-initiated antibiotics group (n = 263,550) | *P* value |
|---|---|---|---|
| Age, years | 77 (67, 84) | 77 (67, 84) | <0.001 |
| Male (%) | 359,096 (56.8) | 161,168 (61.2) | <0.001 |
| Comorbidity, n (%) | | | |
| Malignant tumor | 175,569 (27.7) | 108,257 (41.1) | <0.001 |
| Hypertension | 171,714 (27.1) | 62,954 (23.9) | <0.001 |
| Diabetes mellitus | 133,608 (21.1) | 58,994 (22.4) | <0.001 |
| Heart failure | 113,568 (17.9) | 47,770 (18.1) | 0.047 |
| Cerebrovascular disease | 78,399 (12.4) | 45,007 (17.1) | <0.001 |
| Chronic respiratory disease | 76,772 (12.1) | 21,249 (8.1) | <0.001 |
| Ischemic heart disease | 60,144 (9.5) | 25,524 (9.7) | 0.009 |
| Chronic renal failure | 220,675 (34.9) | 129,788 (49.2) | <0.001 |
| Focus of infection, n (%) | | | |
| Respiratory | 244,676 (38.7) | 69,428 (26.3) | |
| Abdominal | 87,017 (13.8) | 40,363 (15.3) | |
| Urogenital | 49,227 (7.8) | 15,701 (6.0) | |
| Bone and soft tissue | 21,283 (3.4) | 9,426 (3.6) | |
| Meninges/brain/spinal cord | 5,682 (0.9) | 3,013 (1.1) | |
| Heart | 2,268 (0.4) | 1,042 (0.4) | |
| Blood | 386 (0.1) | 283 (0.1) | |
| Hematologic | 41 (0.0) | 29 (0.0) | |
| Others | 65,350 (10.3) | 38,226 (14.5) | |
| Multifocal | 156,812 (24.8) | 86,039 (32.6) | |
| Organ dysfunction, n (%) | | | |
| Respiration | 468,560 (74.1) | 177,881 (67.5) | <0.001 |
| Coagulation | 56,777 (9.0) | 36,632 (13.9) | <0.001 |
| Cardiovascular function | 35,599 (5.6) | 26,229 (10.0) | <0.001 |
| Liver | 27,022 (4.3) | 8,738 (3.3) | <0.001 |
| Renal | 220,675 (34.9) | 129,788 (49.2) | <0.001 |

| | | | |
|---|---|---|---|
| Community-onset sepsis, n (%) | 446,450 (70.6) | 97,368 (36.9) | <0.001 |
| First hospitalization, n (%) | 420,605 (66.5) | 176,030 (66.8) | 0.004 |
| ICU stay, n (%) | 30,838 (4.9) | 23,033 (8.7) | <0.001 |

Data are presented as median (interquartile range).

ICU, intensive care unit

**Supplementary Table 4. Multiple regression analysis for medical cost**

| Variables | Coefficient ($) | 95% CI | *P* value |
|---|---|---|---|
| Short-course antibiotics | -212 | -223 to -201 | <0.0001 |
| Age, per year | -128 | -130 to -125 | <0.0001 |
| Male | 529 | 451 to 608 | <0.0001 |
| community-onset sepsis | -11,287 | -11,369 to -11205 | <0.0001 |
| First hospitalization | 1,680 | 1,598 to 1,762 | <0.0001 |
| Length of hospital stay, per day | 114 | 113 to 115 | <0.0001 |
| ICU stay | 14,436 | 14,273 to 14,600 | <0.0001 |

CI, confidence interval

**Supplementary Table 5. Analysis for 28-day mortality in each focus by cox regression analysis**

**A. Respiratory infection**

| | **Short-course group** | **Long-course group** | **Hazard ratio (95%CI)** |
|---|---|---|---|
| | No. of events / total No. | | |
| Age, years old | | | |
| ≧75 | 5,262/65,257 | 6,400/64,198 | 1.05 (1.01, 1.09) |
| <75 | 2,183/42,692 | 2,882/42,731 | 1.02 (0.96, 1.08) |
| Sex | | | |
| Female | 2,514/40,463 | 2,904/37,986 | 1.07 (1.02, 1.13) |
| Male | 4,913/67,486 | 6,378/68,943 | 1.04 (1.00, 1.08) |
| Community-onset sepsis | | | |
| Yes | 4,150/74,356 | 6,364/72,976 | 0.97 (0.93, 1.01) |
| No | 3,295/33,593 | 2,918/33,953 | 1.25 (1.19, 1.31) |
| First hospitalization | | | |
| Yes | 4,722/75,707 | 5,926/74,703 | 1.05 (1.01, 1.09) |
| No | 2,723/32,242 | 3,356/32,226 | 1.04 (0.99, 1.10) |
| ICU stay | | | |
| Yes | 614/6,128 | 710/6,322 | 0.98 (0.88, 1.09) |
| No | 18253/335285 | 22252/333684 | 1.05 (1.02, 1.09) |

**B. Abdominal infection**

| | **Short-course group** | **Long-course group** | **Hazard ratio (95%CI)** |
|---|---|---|---|
| | No. of events / total No. | | |
| Age, years old | | | |
| ≧75 | 1,235/27,276 | 1,663/28,584 | 0.92 (0.86, 0.99) |
| <75 | 794/26,215 | 1,140/25,593 | 0.85 (0.77, 0.93) |
| Sex | | | |
| Female | 831/21,560 | 1,173/22,944 | 0.94 (0.86, 1.03) |
| Male | 1,198/31,931 | 1,630/31,233 | 0.85 (0.79, 0.92) |
| Community-onset sepsis | | | |

| | | | |
|---|---|---|---|
| Yes | 1,084/32,140 | 1,894/33,255 | 0.85 (0.78, 0.91) |
| No | 945/21,351 | 909/20,922 | 1.08 (0.98, 1.18) |
| First hospitalization | | | |
| Yes | 1,087/35,836 | 1,493/36,943 | 0.92 (0.86, 1.00) |
| No | 942/17,655 | 1,310/17,234 | 0.82 (0.76, 0.90) |
| ICU stay | | | |
| Yes | 99/1,915 | 128/2,159 | 0.93 (0.72, 1.21) |
| No | 1,930/51,576 | 2,675/52,018 | 0.89 (0.84, 0.94) |

**C. Urogenital infection**

| | **Short-course group** | **Long-course group** | **Hazard ratio (95%CI)** |
|---|---|---|---|
| | No. of events / total No. | | |
| Age, years old | | | |
| ≧75 | 784/17,549 | 1,017/18,743 | 1.03 (0.94, 1.13) |
| <75 | 794/26,215 | 1,140/25,593 | 1.02 (0.84, 1.24) |
| Sex | | | |
| Female | 571/14,043 | 738/15,655 | 1.08 (0.97, 1.21) |
| Male | 405/11,898 | 507/10,885 | 0.94 (0.82, 1.07) |
| Community-onset sepsis | | | |
| Yes | 564/16,351 | 887/16,827 | 0.97 (0.87, 1.08) |
| No | 412/9,590 | 358/9,713 | 1.28 (1.11, 1.47) |
| First hospitalization | | | |
| Yes | 577/18,089 | 793/18,731 | 0.95 (0.86, 1.06) |
| No | 399/7,852 | 452/7,809 | 1.12 (0.98, 1.28) |
| ICU stay | | | |
| Yes | 7/170 | 6/157 | 1.30 (0.44, 3.88) |
| No | 940/25,028 | 1,197/25,605 | 1.03 (0.94, 1.12) |

Data are presented as median (interquartile range).

ICU, intensive care unit; CI, confidence interval

**Supplementary Figure 1. Kaplan-Meier curve for 28-day mortality between short and long-course group in the cohort between 2012 and 2016**

There was no significant difference between long-course and short-course (log-rank test, *P*=0.064).

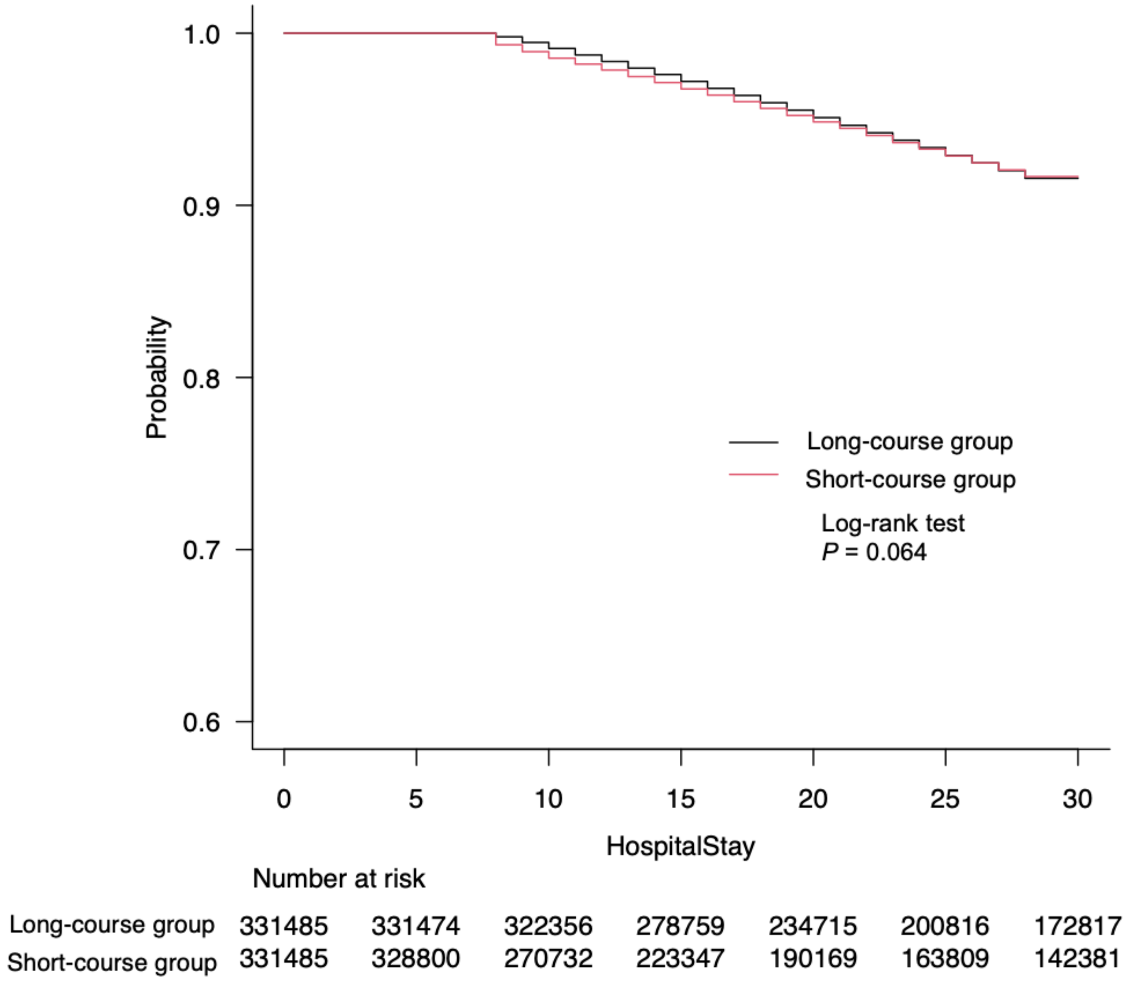

Supplement: Supplementary file 1 — Additional file 1: Table S1. Baseline characteristics. Table S2. Baseline characteristics for sensitivity analysis (2012 to 2016). Table S3. Patient characteristics between non-reinitiated or reinitiated antibiotics group. Table S4. Multiple regression analysis for medical cost. Table S5. Analysis for 28-day mortality in each focus by Cox regression analysis. Figure S1. Kaplan–Meier curve for 28-day mortality between short and long-course group in the cohort between 2012 and 2016 [file 40560_2022_642_MOESM1_ESM.pdf]
